# Precision Oral Medicine: A DPR Segmentation and Transfer Learning Approach for Detecting Third Molar Compress Inferior Alveolar Nerve

**DOI:** 10.1109/JTEHM.2025.3568922

**Published:** 2025-05-12

**Authors:** Yuan-Jin Lin, Shih-Lun Chen, Yi-Cheng Mao, Tsung-Yi Chen, Cheng-Hao Peng, Tzu-Hsiang Tsai, Kuo-Chen Li, Chiung-An Chen, Wei-Chen Tu, Patricia Angela R. Abu

**Affiliations:** Department of Program on Semiconductor Manufacturing TechnologyAcademy of Innovative Semiconductor and Sustainable ManufacturingNational Cheng Kung University 34912 Tainan 701401 Taiwan; Department of Electronic EngineeringChung Yuan Christian University 34900 Taoyuan 32023 Taiwan; Department of Operative DentistryTaoyuan Chang Gung Memorial Hospital Taoyuan 33305 Taiwan; Department of Electronic EngineeringFeng Chia University 34902 Taichung 40724 Taiwan; Department of Electrical Engineering and Computer ScienceChung Yuan Christian University 34900 Taoyuan 32023 Taiwan; Department of Information ManagementChung Yuan Christian University 34900 Taoyuan 32023 Taiwan; Department of Electronic EngineeringMing Chi University of Technology 56082 New Taipei 243303 Taiwan; Department of Electrical EngineeringNational Cheng Kung University 34912 Tainan 701401 Taiwan; Department of Information Systems and Computer ScienceAteneo de Manila University 54724 Quezon 1108 Philippines

**Keywords:** Inferior alveolar nerve, image masking, image segmentation, object detection, third molar, vision transformer

## Abstract

Extraction of the third molar of the mandible is one of the most common oral surgical procedures. Preoperative monitoring and assessment are crucial to mitigate neurological risks. Identifying whether the third molar in the mandible compresses the inferior alveolar nerve still relies on dental professionals, a task that is repetitive and time-consuming. Thus, the primary objective is to utilize dental panoramic radiography for image processing and classify whether the third molar compresses the inferior alveolar nerve, aiming to reduce the demand for CT images in symptom diagnosis and mitigate the risks associated with high-dose radiation. This study proposes an innovative dental panoramic radiography segmentation technique to locate the third molar position. Subsequently, an innovative edge masking enhancement method is used to extract features of the inferior alveolar nerve and the third molar. Moreover, a transformer-based image detection model to consider whether the third molar compresses the inferior alveolar nerve. The third molar position localization method achieved an accuracy rate of 97.92%, compared to recent research at least improved by 3.6% accuracy. Subsequently, innovative edge masking and image enhancement methods improve classification accuracy by 4.3%, when supplemented with computed tomography scan images for further evaluation, the maximum accuracy reached 98.45%, representing a 4.5% improvement compared to previous studies. The third molar position detection results will impact the identification of the inferior alveolar nerve compressed by the third molar. Through the innovative edge region segmentation algorithm can effectively distinguish this object, and the overall evaluation accuracy can be improved by approximately 3.8%.

Clinical Impact –The development of an image enhancement-based classification model makes it easier to predict the likelihood of the third molar compressing the inferior alveolar nerve during dental panoramic X-ray examinations. Additionally, early detection of potential issues helps prevent nerve damage, reduces exposure to computed tomography scans, and provides dentists with objective treatment assessments, allowing for more communication time with patients.

## I. Introduction

The third molars are the last molars to emerge in the oral cavity and commonly known as “impacted teeth,”. They are typically located at the very back of the mouth, with one in each of the upper and lower quadrants, usually erupting during late adolescence or early adulthood (between the ages of 17 and 25). Third molars are generally classified based on their position and degree of eruption. Common classification methods include the Pell and Gregory classification [1] and the Winter classification [2]. The Pell and Gregory classification is based on the relationship between the occlusal surfaces of the second and third molars and the ascending ramus of the mandible. On the other hand, the Winter classification categorizes according to the vertical position and degree of impaction of the third molars, including horizontal impaction, vertical impaction, and transverse impaction. Additionally, the relationship between the third molars and the inferior alveolar nerve (IAN) is often discussed [3], [4], as it is used to assess the depth of mandibular third molars and their relative position to the ascending ramus. As a result, the eruption status of third molars can lead to various symptoms, such as periodontal disease, dentigerous cysts, and tumors [5]

**FIGURE 1.**
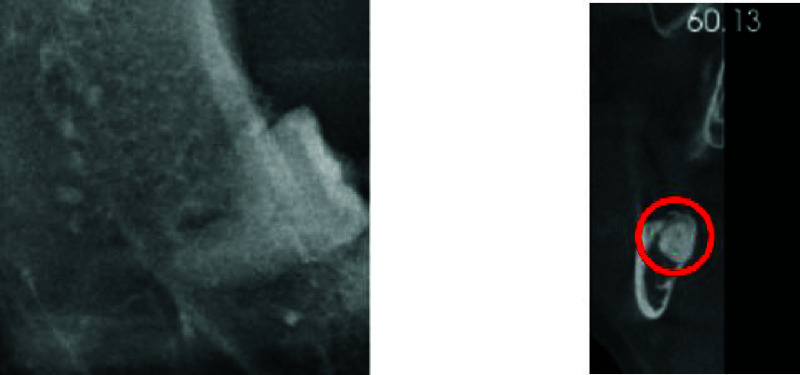
The example of third molar compress IAN.

In traditional dental practice, patients suffering from conditions caused by the eruption of the mandibular third molars are typically advised by dentists to undergo third molar extraction surgery. This is one of the most common oral surgical procedures. Third molars are typically examined using Dental Panoramic Radiography (DPR) and computed tomography (CT). The DPR and CT images of the third molars and IAN are illustrated in Fig. 1. The standard procedure begins with DPR to assess the morphology of the third molars and their relationship with surrounding critical structures. For the maxillary third molars, the relative position to the sinus must be considered, while for the mandibular third molars, the proximity between the tooth roots and the IAN is crucial. If the roots are close to the IAN, it is recommended to perform a 3D dental CT to accurately determine the position of the roots relative to the nerve, thereby reducing the risk of postoperative nerve damage [4]. However, considering the significant increase in patient costs and radiation exposure associated with CT [6], [7] many studies have explored the use of image processing and deep learning techniques on dental X-ray images to solve this problem [8], [9], [10]. Artificial intelligence-assisted diagnostic techniques in dentistry can currently be categorized into three types: image classification, object detection, and instance segmentation [11]. Convolutional Neural Networks (CNN) are among the most prominent algorithms in the field of deep learning [12]. CNNs effectively classify dental conditions by extracting features from images [13], [14], [15], [16], [17]. Moreover, CNN-based object detection models are usually used in the field of dental-assisted diagnosis, such as for multi-condition detection in DPR [18], detection of dental calculus in Bitewing Radiographs [19], and instance semantic segmentation techniques are applied for dental plaque detection [20].

Extensive research has been conducted on the relationship between the IAN and third molars, achieving significant results using various deep learning architectures. For instance, combining the MobileNet-v2 architecture with skeletonization algorithms and the signed distance method resulted in an accuracy of 0.943, a sensitivity of 0.941, and a specificity of 0.800 [12]. Another study utilizing a ResNet-50-based image classification model achieved an accuracy of 80.65% [21]. A deep learning network architecture based on YOLOv4 was used to assess the relationship between the IAN and third molars, achieving an accuracy of 88.77% and a recall rate of 91.67% [22]. Additionally, using GoogLeNet with adjusted input image sizes yielded an accuracy of 0.92, a sensitivity of 0.874, and a specificity of 0.952 [23]. The use of RetinaNet and YOLOv3 for predicting DPR images resulted in a sensitivity of 82.02% and a specificity of 75.28% [24]. Despite advancements in artificial intelligence (AI), directly applying AI models to DPR to assess the relationship between the IAN and the third molar remains challenging. The key challenges are as follows:

### A. Complexity and Diversity of DPR Image

Imaging angles, anatomical variations among patients, and noise interference influence the resolution, contrast, and overall quality of DPR images. These factors can significantly impact the robustness and diagnostic accuracy of AI models. Accurate localization of the third molar and its relationship with the IAN is not solely dependent on the extraction and classification of single features. It requires a comprehensive analysis of multiple imaging features, such as tooth boundary clarity, nerve canal visibility, and image enhancement techniques. However, conventional CNN-based models rely on local feature extraction, which may overlook the long-range spatial relationships between the IAN and the tooth roots, leading to inaccurate assessments. In contrast, Transformer-based models through their self-attention mechanism, can simultaneously consider different regions of the image, improving the identification of IAN compression risks [25], [26].

### B. Limitations of CNN-Based Classification Model

CNNs often suffer from overfitting and may lose critical spatial information due to the high variability of DPR images, ultimately reducing their generalization capability. The limited receptive field of CNNs may lead to learning spurious correlations, such as image borders or background noise, rather than the clinically relevant features that impact IAN compression risk. In contrast, Transformer-based models equipped with multi-head self-attention mechanisms can capture the global relationships between the tooth and the IAN, preventing misclassification caused by CNNs’ reliance on localized features. Additionally, Transformers exhibit superior cross-domain generalization, enabling them to adapt to images acquired from different devices and medical institutions, enhancing their clinical applicability. For instance, Zhou et al. [27] applied the Swin-Transformer to classify pathological features in pediatric DPR images for caries detection. Compared to the traditional CNN-based ResNet model, and improved classification accuracy by 5%, demonstrating the superior adaptability of transformer-based architectures in dental image analysis. This further highlights the potential of transformers to overcome the limitations of CNNs in handling diverse medical imaging conditions, making them a promising approach for IAN risk assessment in DPR images.

### C. Practical Clinical Needs in Dentistry

Existing approaches often fail to address the broad spectrum of clinical diagnostic requirements, as most AI models are trained on small-scale or non-open-source medical datasets, limiting their application to private hospitals or specific healthcare facilities. Additionally, variations in image quality, disease severity, and the presence or absence of image normalization may affect the reliability of AI-based assessments in real-world clinical settings. Therefore, to ensure adaptability across diverse clinical environments, this study employs a Transformer-based imaging analysis approach to overcome the limitations of CNN-based models. Furthermore, this study integrates the Tufts open-source dental database [28] for evaluation, ensuring that the model can adapt to DPR images from multiple medical institutions, enhancing diagnostic reliability and practical usability.

Therefore, this study aims to develop an algorithm and a deep learning-based assistive system to enhance the efficiency of dentists and reduce patient waiting times. This study’s edge region segmentation and edge masking techniques are designed to help evaluate the relationship between the mandibular third molar and IAN, particularly in feature extraction involving the tooth and adjacent structures. By leveraging these innovative techniques, the proposed classification model improves robustness and reliability, reducing misdiagnosis and minimizing unnecessary CT scans, thereby decreasing patient radiation exposure. Furthermore, Transformer-based techniques have not yet been explored for diagnosing the compression of the IAN by the third molar in DPR images. Given their superior ability to model global spatial relationships, we hypothesize that incorporating Transformer architectures could enhance diagnostic performance in this field. This study also evaluate ConvNeXt [29] and compares its performance against conventional CNN models to assess the effectiveness of different deep learning paradigms in DPR image analysis. The goal is to address the challenges posed by the similar pathological features of third molars and the difficulty in observing them, thereby alleviating the diagnostic burden on dentists. The system architecture can be divided into several main components: DPR segmentation techniques developed in this study are used to extract the mandibular third molar and the associated IAN. Next, the edges of the mandibular third molar crown are captured for bilateral masking and image enhancement. Finally, the system evaluates the images using a pathological feature classification model. The primary contributions and novelty of this study are as follows:

1. This study develops a novel Edge Region Segmentation Algorithm for DPR segmentation, achieving an accuracy of 97.92%. Our approach offers higher precision and reduced inference time than other YOLO models.
2. Adopting an innovative Edge Mask Enhancement Algorithm and image masking techniques for detecting third molar compression on the IAN improves classification accuracy by 4.3%, with a maximum accuracy of 98.45%.
3. A Transformer-based model is employed for pathological feature detection, demonstrating higher accuracy, shorter inference time, and better generalization than traditional CNN-based models.
4. Integrating a Fuzzy Voting mechanism ensures reliability in the final model validation, reducing misclassification risks, and the validated result can reach 97.44%.
5. In the final comparative analysis, ConvNeXt_v2 achieves the highest accuracy of 98.45%, representing an improvement of approximately 4.5% over existing studies.

The structure of this article is as follows: The first part introduces the research background and previous related work of this study. In the second part, the study discusses and proposes groundbreaking strategies. The third part presents the experimental results and their evaluation. The fourth part compares the strengths and weaknesses with previous studies and discusses the limitations of this research. Finally, the fifth part summarizes this work and proposes future developments.

## II. Methods

**FIGURE 2.**
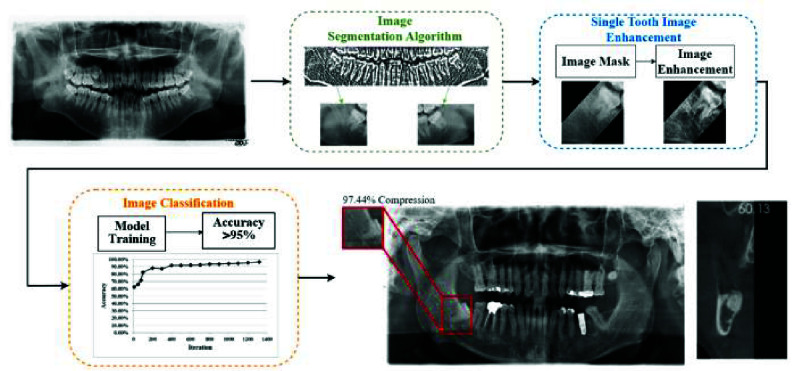
The flowchart for the third molar and IAN relationship detection.

To alleviate the diagnostic burden on dentists and reduce the use of high-radiation CT scans, this study proposes an auxiliary assessment method based on image processing and deep learning for evaluating the eruption status of the third molar and its potential impact on the IAN. The study can be separated into three stages: first, using image segmentation techniques to identify the mandibular third molar in DPR images; next, applying image masking and enhancement techniques to extract features of the IAN and mandibular third molar; finally, training the enhance single tooth image dataset for disease detection and assisting diagnosis with CT images. The overall workflow of the study is illustrated in Fig. 2.

### A. Dataset Collection

The database used in this study was collected and annotated by dentists with over five years of experience. Clinical DPR and CT images provided by Chang Gung Memorial Hospital in Taoyuan, Taiwan, served as the source of training images for this research. Detailed information about the database is shown in Table 1. The images used in the study are covered under IRB protocol number: 02002030B0, and all images used were obtained with patient consent. The study selected DPR images containing at least a third molar as the training dataset. Data augmentation techniques were applied to the DPR images, including vertical flipping and brightness adjustment. The DPR images’ height was 1540 pixels, and the width ranged between 2700 and 2900 pixels. The resulting single-tooth dataset was randomly divided into training and testing sets at a 7:3 ratio, with 70% allocated to the training dataset and 30% to the testing dataset. CT images are used as a reference for validation and comparison without further processing. After database augmentation, 3,295 third molar images are available for training the image classification model r. The trained model was evaluated based on its ability to determine whether the third molar impinges on the nerve and assess the third molar’s eruption status. In addition to the DPR dataset used in this study, the Tufts Dental Database [28] provides 1,000 expert-annotated DPR images publicly available for research. This study randomly selected DPR images from the Tufts dataset to validate the model’s Robustness and Generalization.

**TABLE 1:** DPR and ct dataset provide by chang gung memorial hospital


Category	Number
DPR	1073
CT	16
Third Molar tooth	3295

### B. DPR Region Segmentation

**FIGURE 3.**
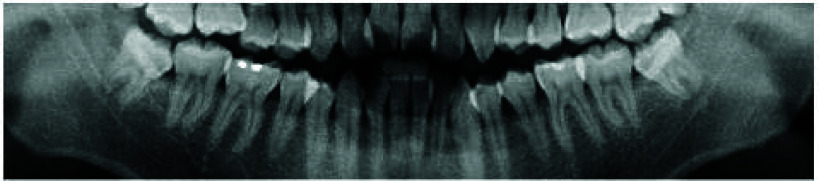
The DPR partial mask result.

The original DPR is resized to a uniform target dimension of $1540\times 2816$ to help mitigate variability resulting from differences in image sizes. Image segmentation technology extracts the maxilla and mandible regions from the entire image. Specific rows and columns are selected to encompass the lower teeth of interest. A mask is generated at 70% of the image height by masking techniques to emphasize the maxilla and mandible regions [31], with pixel values inside the mask set to 0 (white) and pixel values outside the mask set to 1 (original image). The pixels within the masked area are retained, and those outside the mask are white, enhancing the visibility of the maxilla and mandible regions. Applying the mask to the original DPR yields a masked image where the maxilla and mandible regions are easily identified and evaluated, as shown in Fig. 3.

#### 1) Grayscale Transformer

Due to each mandibular image being located at a different location and affected by various environmental factors, this problem may result in varying degrees of grayscale contrast changes after masking. This study adopted grayscale transformation to highlight the contrast between the background and teeth and adjust the image’s pixel values. By adjusting the image’s grayscale brightness values, this technique simplifies the representation of all pixels in the image.

#### 2) Gaussian High Pass Filter

**FIGURE 4.**
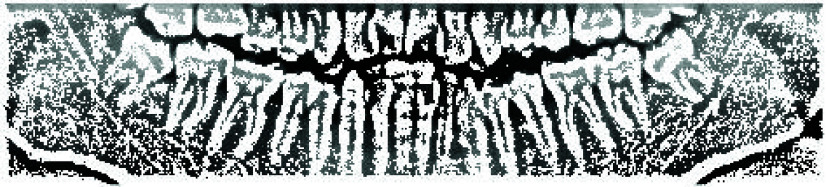
The Gaussian high pass filter image.

One of the primary challenges in symptom analysis is the presence of noise points in the images. Therefore, applying filtering techniques to reduce these unnecessary noise signals is crucial. The Gaussian high pass filter is a common image enhancement and feature extraction technique. The Gaussian high-pass filter is the most suitable choice to emphasize tooth edge features. It is represented as (1), where D0 is the cutoff frequency, and D(u,v) is the distance from the center of the frequency rectangle. As D0 increases, the smoothness improves. Since the IAN belongs to the detailed part of the image, this study sets D(u,v) to 10 and D0 to 1. This allows for smoothing the image while preserving the details, as shown in Fig. 4.

\begin{equation*} \mathrm {H(u,v)=}e^{-D^{\mathrm {2(}u,v\mathrm {)/2}D_{0}^{2}}} \tag {1}\end{equation*}

#### 3) Contrast Stretching

**FIGURE 5.**
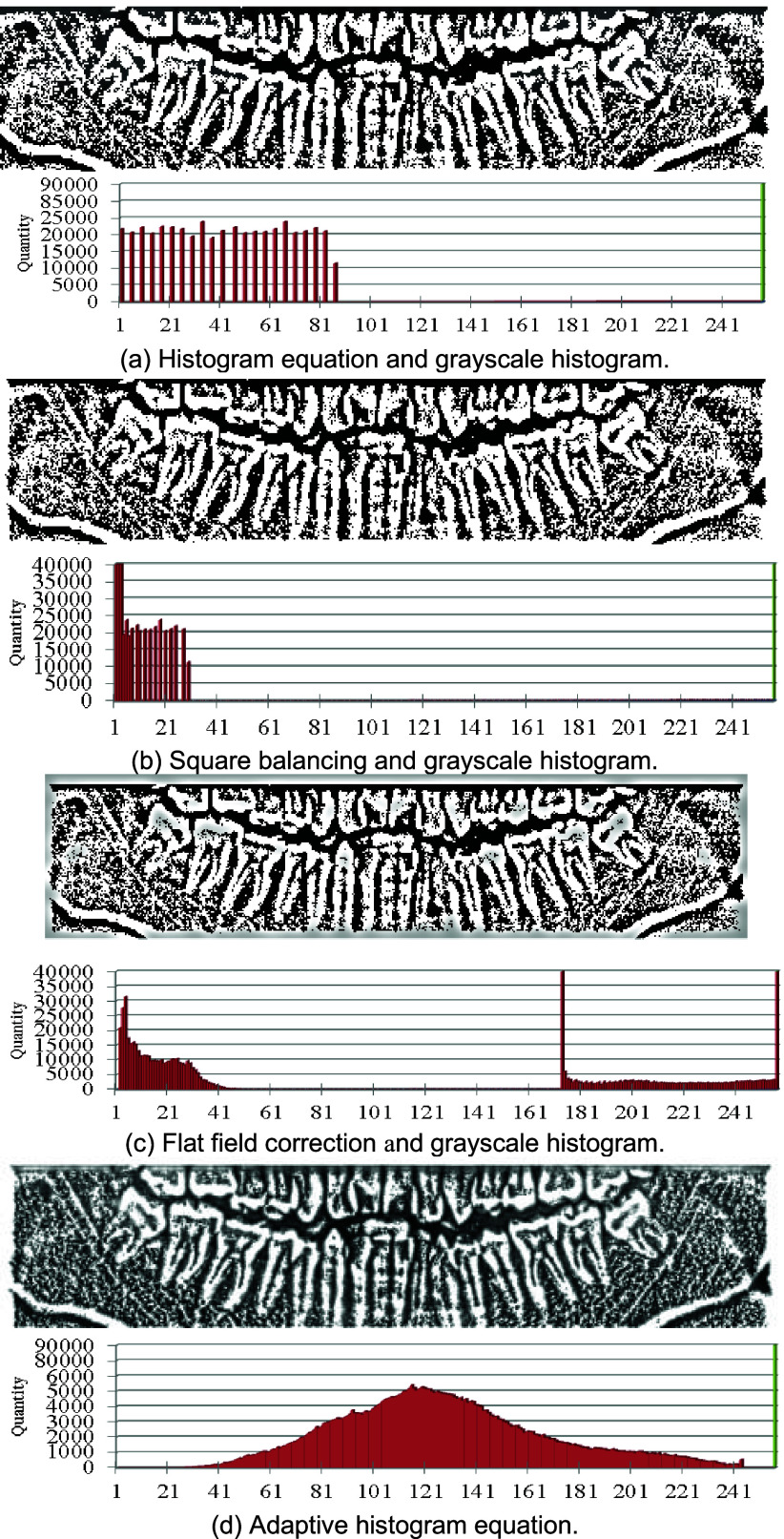
Result of contrast stretching and grayscale histogram.

Contrast stretching is a widely used technique in image processing aimed at increasing the contrast by expanding the brightness distribution of the image. Contrast stretching aims to enhance features by increasing the image contrast. Contrast enhancement through histogram equalization is a method that redistributes the brightness levels of the image, making the brightness levels more uniformly distributed, thereby increasing contrast, as shown in Fig. 5(a). After histogram equalization, the square balancing technique enhances the differences between different brightness levels. Subsequently, the image undergoes min-max normalization to map pixel values to the range of 0 to 255 for display purposes, as depicted in Fig. 5 (b).

Flat-field correction is employed to mitigate uneven brightness points in the image, ensuring that pixel values tend toward uniformity. This correction helps to eliminate bright differences caused by uneven illumination. Flat-field correction adjusts the brightness and contrast of different regions in the image, as illustrated in Fig. 5 (c). This study adopts the histogram equalization technique to tweak how the image looks based on local regions of the image. Essentially, this method adjusts the contrast to meet the specific needs of different areas within the image, by carefully setting the parameters. This study ensures that adaptive histogram equalization preserves the details in the image and avoids excessive enhancement. It can be seen the results of these adjustments are in Fig. 5 (d), which clearly shows the image remains balanced.

This study includes grayscale cumulative distribution plots for each step. From the histogram equalization to the flat-field correction steps, the grayscale values bunch up at the extremes. This happens because the original image has a minimal range of intensity values—most pixels are either white (255) or completely black (0). This leads to almost no pixels with mid-range values. Therefore, even though histogram equalization tries to spread out the pixel intensities to have an even number of pixels at each intensity level, the mid-range pixels still end up practically nonexistent. However, when it comes to the adaptive histogram equalization step, more values are in the middle range. This process breaks the image down into smaller sub-regions, computes a histogram for each one, and then redistributes the pixel intensities according to these individual histograms. This tailors the pixel intensity redistribution to fit the specific characteristics of each sub-region, leading to more pixels with intermediate intensity values, especially in the darker areas of the image.

#### 4) Binarization and Morphological Operations

**FIGURE 6.**
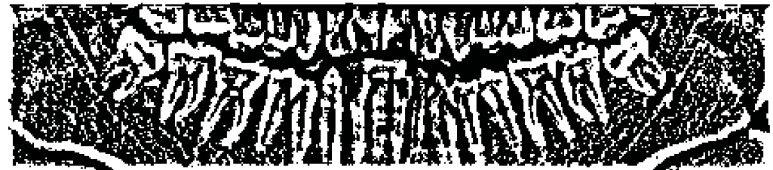
DPR binarization result.

**FIGURE 7.**
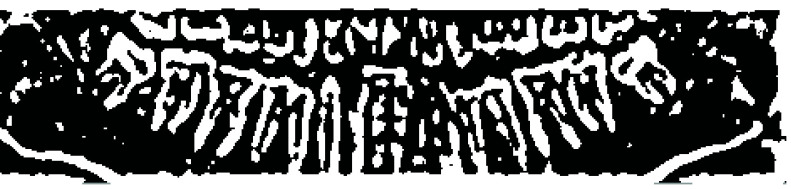
DPR binarization image after median filter.

**FIGURE 8.**
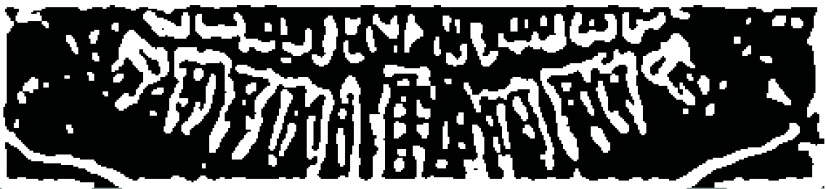
DPR binarization image after open operation.

Binarization technique is a method in image processing [32], which converts a grayscale image into a binary image, effectively highlighting the brightness of the IAN. This study utilizes the commonly used Otsu’s binarization method to select a threshold to separate image pixels into foreground and background. According to data analysis, the adaptive threshold mean M for this study is 0.69, and the standard deviation $\sigma $ is 0.011, as shown in (2) and Fig. 6. This method efficiently converts the image into a binary image, as illustrated in Fig. 6. Morphological operations primarily deal with the shape, structure, and geometric features [33]. In this study, median filtering is first applied to the image to reduce noise, as shown in Fig. 7. The opening operation is applied to smooth the image and remove unnecessary information depicted in Fig. 8.

\begin{align*} \sigma \sigma I\left ({{ x,y }}\right )=\begin{cases} \displaystyle \mathrm {0, }\quad if~I\left ({{ x,y }}\right )< M\mathrm {+0.5} \\ \displaystyle \mathrm {255, }\quad if~I\left ({{ x,y }}\right )\ge M\mathrm {+0.5}  \end{cases} \tag {2}\end{align*}

#### 5) Vertical Grayscale Projection

**FIGURE 9.**
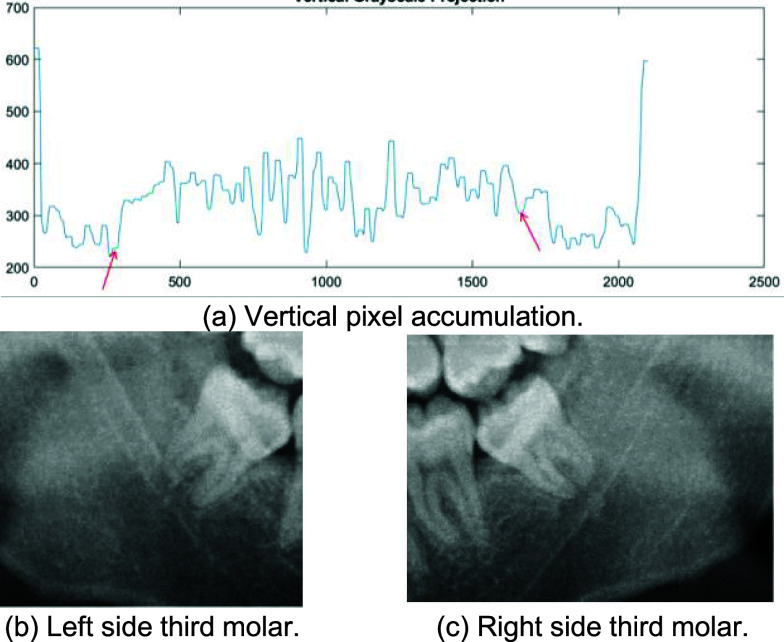
Vertical grayscale projection.

This study presents a tooth localization method utilizing vertical grayscale projection to roughly segment teeth in DPR. Vertical grayscale projection is a technique used to analyze the brightness distribution in different regions of an image as shown in Fig. 9 (a). The approximate third molar positions are determined based on the valleys of the projection distribution. The teeth are according to the segmented criterion in the first valley where the amplitude on one of the left and right sides exceeds 10 units, as depicted in Fig. 9 (b), (c).

#### 6) Edge Region Segmentation

**FIGURE 10.**
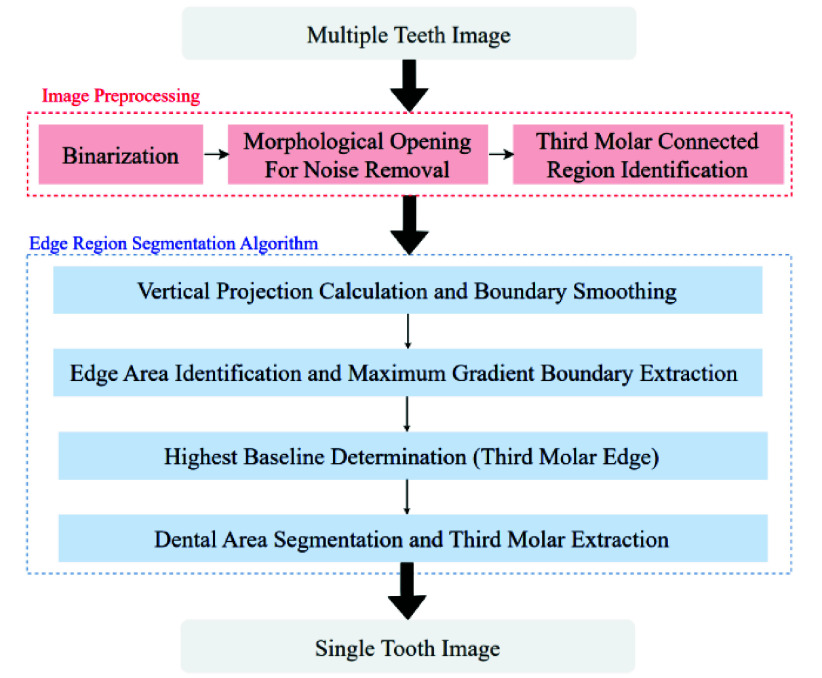
Edge Region segmentation flowchart.

As the vertical grayscale projection only roughly locates the area of a single tooth and cannot precisely identify the position of the third molar, this study develops a novel edge region segmentation algorithm based on the vertical grayscale projection localization method. This algorithm aims to automatically locate the position of the third molar and the IAN. The edge region segmentation algorithm is a technique used to analyze the distribution of different connected regions in an image, and the algorithm flowchart is shown in Fig 10.

**FIGURE 11.**
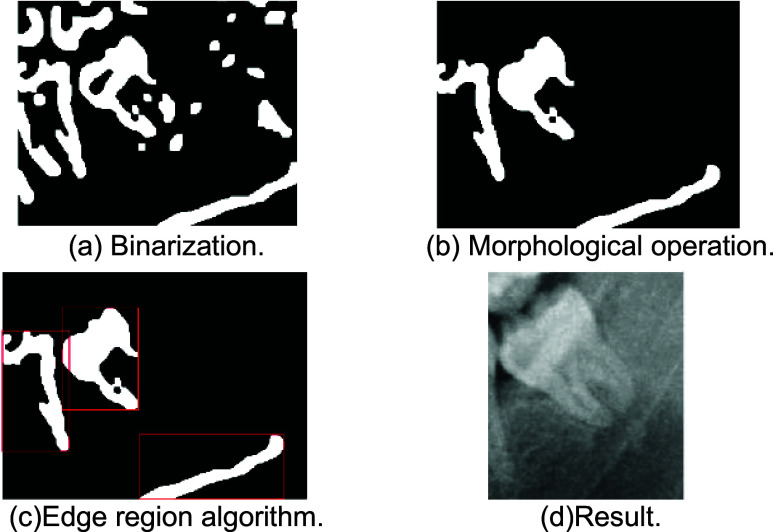
The result of edge region algorithm.

In this subsection, the tooth image segmented by the vertical grayscale projection method is binarized, as shown in Fig. 11 (a). Through morphological operations and other denoising methods, the connected region of the third molar is identified, as shown in Fig. 11 (b). Subsequently, all edge regions of the connected are identified and displayed, as shown in Fig. 11 (c). The edge region segmentation algorithm also can identify the edge region position of the third molar. First, find the bottom lines of all edge regions. Taking Fig. 11 (c) as an example, find out the bottom lines of the three red bounding boxes. Then, Find the highest bottom line in this image, and this bottom line is the edge region of the third molar. The reason for choosing bottom line is that the third molar is not necessarily the tallest object. Therefore, the tooth region is divided based on the edge region selected by the bottom line. The result is shown in Fig. 11 (d).

### C. Tooth Mask Enhancement

**FIGURE 12.**
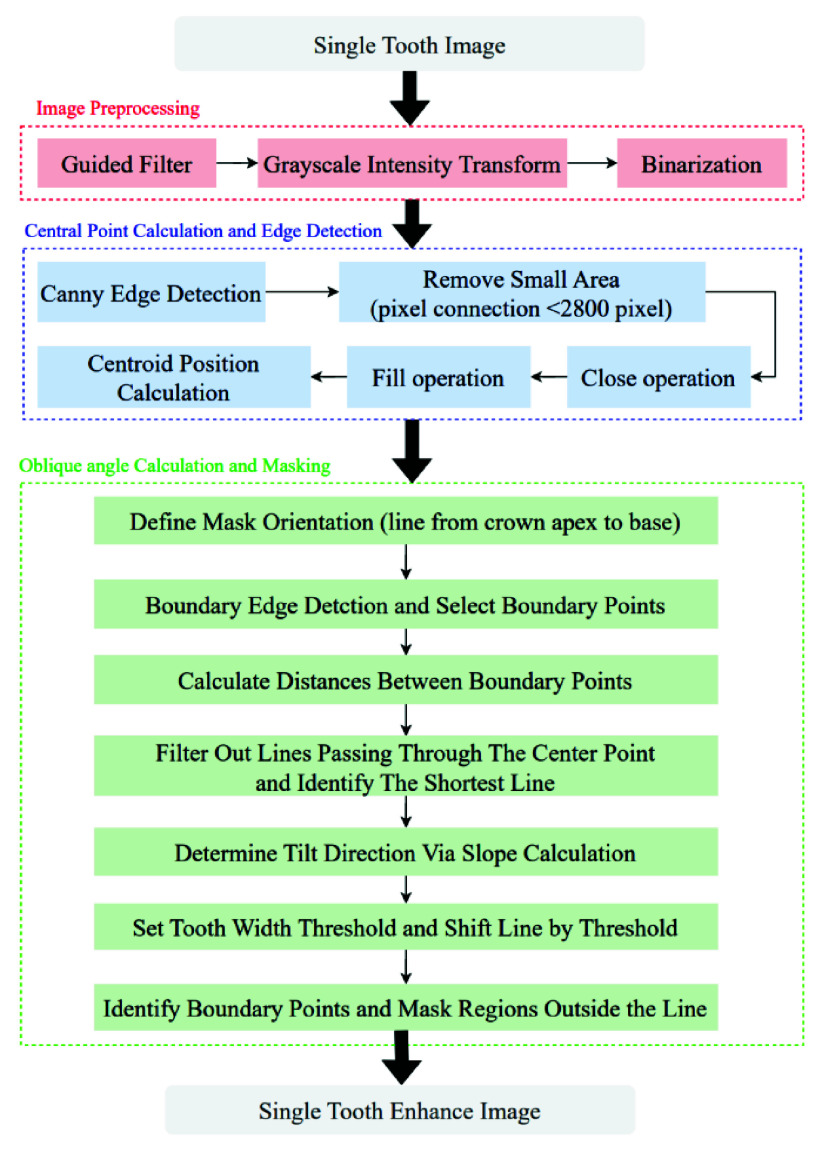
Edge Masking enhancement flowchart.

This study employs image enhancement techniques to remove most noise from third molar images and improve the classification accuracy of the third molar and IAN features. An image masking technique based on tooth crown edges is also applied to exclude non-third molar regions, with the enhancement method detailed is shown in Fig. 12.

#### 1) Third Molar Image Pre-Processing

**FIGURE 13.**
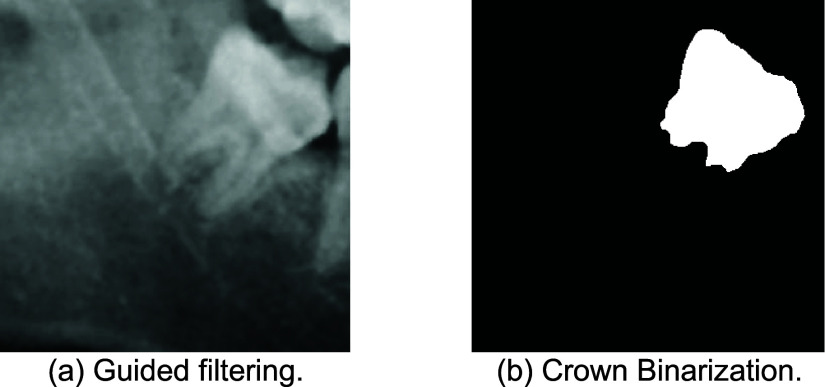
The result third molar image pre-process.

Guided filtering is a specific type of filter used in image processing to preserve details and structures while smoothing the image and incorporating guided information. This study uses the NeighborhoodSize method to control the size of the rectangular neighborhood around each pixel used in the specified guided filtering. The filtering results are shown in Fig. 13 (a). This study calculated the average pixel value of the third molar images after image preprocessing, representing the brightness level of the entire image. Different brightness adjustments are made to each image based on the calculated average pixel value. If the average pixel value is lower than the threshold, the bright range is adjusted to the brighter area, which helps to highlight the brightness of the target areas and make it easier to identify. Subsequently, the processed image is converted into a binary image using a bright threshold. In this image, pixels with brightness above the threshold are considered as target areas (white areas), while pixels with brightness below the threshold are considered as background. The brightness image adjustment threshold method in this study is shown in (3), where CS is Contrast Stretching, MPV is mean pixel value, and different Contrast Stretching can be performed according to four intensity levels from small to large thresholds.

\begin{align*} CS= \begin{cases} \displaystyle 1,~if \quad 0\le \mathrm {MPV< 122} \\ \displaystyle 2,~if\quad 122\le \mathrm {MPV< 130} \\ \displaystyle 3,~if\quad 130\le \mathrm {MPV< 140} \\ \displaystyle 4, ~if\quad 140\le \mathrm {MPV}\le 255  \end{cases} \tag {3}\end{align*}

This study utilizes regionally connected area filtering to eliminate the numerous small noises in the binary image other than the third molar. This technique extracts objects from the binary image by analyzing the size of their connected areas, preserving only regions with a connected area exceeding a set threshold. This helps ensure that the remaining areas are primarily the target areas (teeth) rather than small noises. This method generates a cleaner binary image where white pixels represent the areas of interest and black pixels represent the background, as shown in Fig. 13 (b).

#### 2) Central Point Calculation and Edge Detection

**FIGURE 14.**
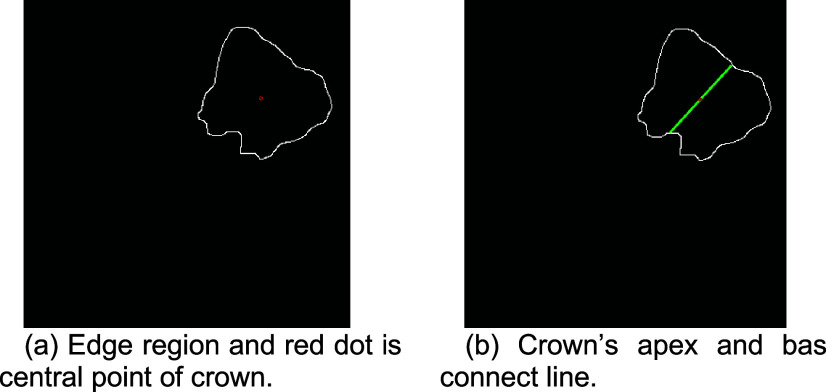
The third molar central point process.

Edge detection is employed to accurately determine the positioning of subsequent masks to identify the contours of the third molar edge, serving as the basis for setting the mask’s position. The Canny edge detection method is chosen for its superior accuracy in capturing finer details. This method identifies edge positions by computing gradients within the image and retains only the pixels with local maximum gradients. The Canny method implies that only pixels with the highest gradients are considered edges, while other pixels are suppressed. The process begins with smoothing the image using a Gaussian filter to reduce noise interference and enhance detection accuracy. Computed centroids and contours are plotted on the edge regions to visualize the positions and shapes of the target objects within the image, are shown in Fig. 14(a).

#### 3) Oblique Angle Calculation and Masking

This study identifies the tilt direction of the third molar to ensure the accuracy of mask orientation, typically defined as the line connecting the crown’s apex to its base, with the slope of this line representing the tilt direction. The study employs edge detection techniques to select boundary points within the edge regions, calculate the distances between each boundary point, and filter out all lines passing through the center point. Finally, the shortest line, as depicted in Fig. 14 (b). This shortest line represents the connection between the crown’s apex and base. The tilt direction of the third molar can be identified by determining the slope of this line.

**FIGURE 15.**
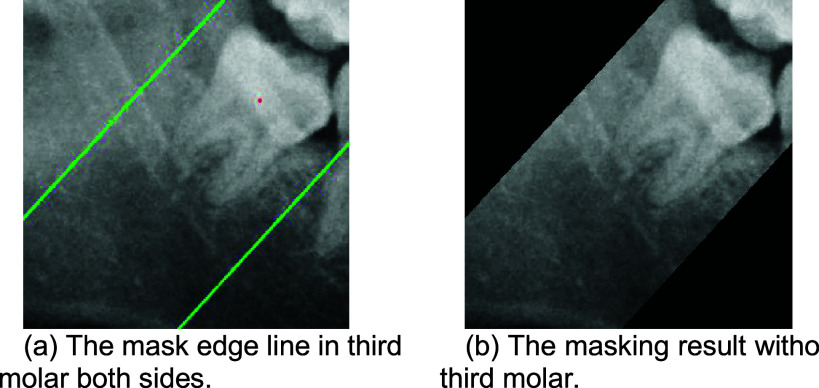
The result of third molar masking.

After determining the tilt direction of the third molar using the slope of the line, this study sets a threshold value related to the width of the tooth. By moving the line from the center point to both sides by this threshold distance, the boundary points of the third molar can be identified, as shown in Fig. 15(a). Subsequently, the regions outside the line are masked with black, as depicted in Fig. 15 (b).

#### 4) Third Molar Image Enhancement

**FIGURE 16.**
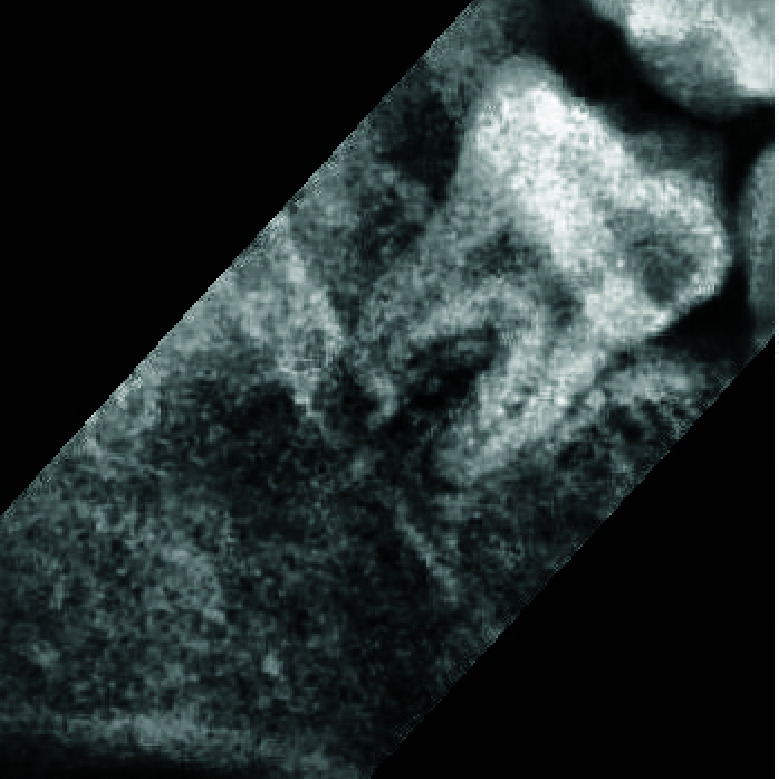
The result of image enhancement.

After applying the edge masking to the third molar, this study utilized various image enhancement techniques to clarify the relationship between the third molar and the IAN. The training results using different image enhancements which is shown in TABLE 3. Contrast stretching proved effective in making the IAN brighter and more prominent while darkening its surroundings, as illustrated in Fig. 16.

**TABLE 2:** Classification model parameter setting

Parameter		Value		Parameter	Value
Learning Rate		0.0001		Learning Rate Drop Factor	0.1
Batch Size		16		Learning Rate Drop Period	10
Shuffle		Every-epoch		Validation Frequency	10
Parameter: Epoch					
Model	Alex Net	Google Net	Shuffle Net	Mobile Net_v2	Conv NeXt_v2
Value	30	30	20	25	15

**TABLE 3:** Comparison with YOLO and Algorithm in different evaluation metrics

Model	YOLOv5	YOLOv7	YOLOv8	YOLOv10	ERSA
Accuracy	82.75%	91.93%	93.98%	95.86%	97.92%
Precision	88.31%	95.66%	95.85%	95.54%	96.59%
Sensitivity	87.90%	97.42%	98.91%	98.38%	97.86%
Specificity	88.42%	93.60%	91.76%	95.62%	96.12%
F1-score	88.10%	96.53%	97.35%	96.93%	97.22%
mAP50	85.51%	96.78%	99.02%	97.42%	97.23%
Inference Time (sec)	1.43	1.64	1.71	1.32	1.074
Memory Usage	78.49%	86.32%	83.85%	81.95%	18.14%

### D. Image Classification & Object Detection

**FIGURE 17.**
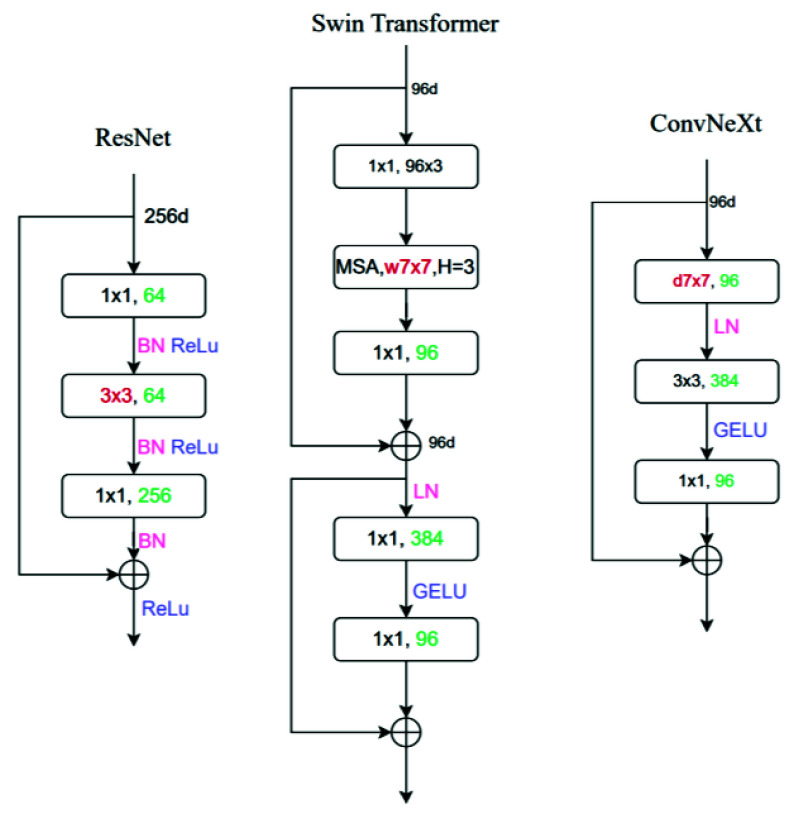
ConvNeXt evolution and micro-level model design.

CNN is one of the earliest architectures and is widely applied in deep learning, achieving remarkable success in tasks such as image classification and object detection through automatic feature extraction in convolutional layers. With technological advancements, the YOLO series introduced an end-to-end real-time object detection method, further advancing the application of deep learning in image processing. Due to its efficiency and accuracy, YOLO has become one of the mainstream methods for object detection. However, with the emergence of the Transformer architecture, the deep learning community began to recognize its potential in image processing. Originally developed for natural language processing, the Transformer’s powerful self-attention mechanism has also demonstrated exceptional performance in tasks such as image classification and object detection. The advent of Vision Transformer (ViT) has gradually challenged the dominance of CNN in the image processing field. Against this backdrop, ConvNeXt_v2 was developed, combining the strengths of traditional convolutional neural networks and Transformer architectures. Based on the ResNet architecture, ConvNeXt_v2 simplifies the complexity of modern Transformer architectures by introducing several key improvements: replacing ReLU with GeLU, reducing the number of activation functions and normalization layers, substituting Batch Normalization with Layer Normalization, and decoupling the downsampling layers from ResNet. These modifications make the model lightweight and efficient. The modified architecture is illustrated in Fig. 17.

Furthermore, this study provides the parameter settings for training different classification models, as shown in Table 2. The selected parameters include learning rate of 0.0001, batch size of 16, learning rate drop factor of 0.1, learning rate drop period as 10, shuffle setting as every epoch, and validation frequency as 10. Additionally, we have defined the maximum training epoch for each model to ensure complete convergence during training.

To evaluate the model training efficacy, different standards are typically used for comparison. Commonly used standards in current research as shown in below, and calculated based on the confusion matrix, with formulas as illustrated in (4), (5), (6) and (7).

1. Precision: The proportion of all items detected as targets that are correctly classified as targets.
2. Recall: The proportion of all targets in the data that are correctly classified as targets, also call as sensitivity.
3. mAP (mean Average Precision): The average of these average precision values across all classes, which is computed by plotting a precision-recall curve for each class and calculating the area under the curve (AUC).
4. Specificity: The proportion of targets that are actually no diseases are tested as correct.

\begin{align*} Precision& = \frac {T_{p}}{T_{p}+F_{p}} \tag {4}\\ Recall(\mathrm {Sensitivity})& = \frac {T_{p}}{T_{p}+F_{n}} \tag {5}\\ mAP& = \frac {\sum \nolimits _{q=1}^{Q} {\frac {1}{n}\sum \nolimits _{r^{I}\{0,0,1\ldots \ldots 1\}} {P_{interp}(q)}}}{Q} \tag {6}\\ Specificity& =\frac {T_{N}}{F_{P}+T_{N}} \tag {7}\end{align*}

## III. Result

This section can be divided into two parts. The first part focuses on exploring the effectiveness of using different image processing for DPR segmentation and comparing them with the image segmentation technique. The second part involves the evaluation of the image classification model, where the training results of different classification models are assessed with validation using CT images to ensure whether the third molar in DPR genuinely compresses the IAN.

### A. DPR Segmentation Evaluation

**FIGURE 18.**
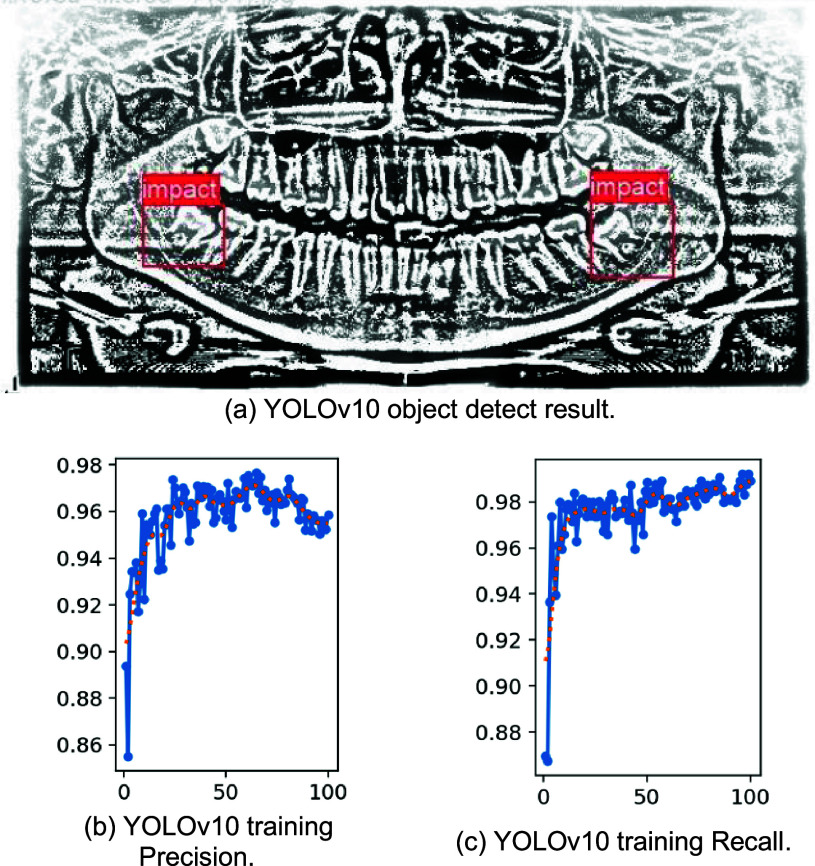
The training and validation result use YOLOv10 to detect third molar and IANs.

YOLO is a series of object detection models that have gained widespread attention due to their simplicity, speed, and accuracy. The YOLO series includes YOLOv5, YOLOv7, YOLOv8, and YOLOv10. The objective of this stage is to extract the third molar and IAN from DPR. To achieve this goal, two approaches were used: YOLO and the edge region segmentation algorithm employed to locate the third molar and IAN. First, DPR was subjected to high pass filtering to enhance the features of the third molar and IAN. Then, YOLO was used to identify the third molar compress IAN in DPR, as shown in Fig. 18(a). The training precision and recall are presented in Fig. 18 (b), Fig. 18 (c). Table 3 shows YOLOv10 achieves an accuracy of 95.86%, with a precision of 95.54%, a sensitivity of 98.38%, and a specificity of 95.62%. The second-best performing model, YOLOv8, achieved an accuracy of 93.98%, with precision, sensitivity, and specificity reaching 95.85%, 98.91%, and 91.76%, respectively. On the other hand, the proposed Edge Region Segmentation Algorithm (ERSA) developed in this study achieved a notable accuracy of 97.92%, with a precision of 96.59%, a sensitivity of 97.86%, and a specificity of 96.12%. Compared to previous methods, this study demonstrates an improvement of at least 2% to 6% in segmentation accuracy.

### B. Third Molar Compress Ian Classification

**FIGURE 19.**
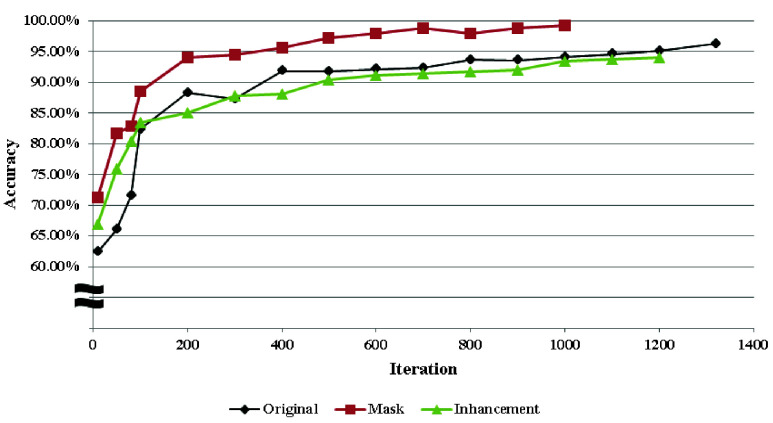
ConvLeXt_v2 training process with different method.

This study evaluates the classification of whether the third molar compresses the IAN using five different classification models. The training processes based on various methods are shown in Fig. 19. ConvNeXt_v2 is employed as the image classification model for training. It can be observed that the training results using only image enhancement are approximately 2% lower than those using original images, and other image enhancement methods are shown in Table 4. However, better results can be achieved by combining masking and enhancement methods. Table 5 presents the training process of ConvNeXt_v2 with image enhancement and masking methods.

**TABLE 4:** Image enhancement training result

Method	Accuracy
Contrast stretching	96.27%
Histogram equalization	94.41%
Linear transformation	80.88%
Contrast stretching & Linear transformation	84.41%
Presence of noise & Contrast stretching	90.59%
High-pass filter	91.47%

**TABLE 5:** Convnext_V2 training process using image enhancement and image masking

Epoch	Iteration	Training time	Training	Testing
1	1	00:00:10	51.50%	51.63%
5	150	00:02:55	87.66%	92.83%
10	330	00:06:18	97.41%	94.42%
15	510	00:11:31	98.12%	97.21%
20	690	00:17:49	97.99%	98.10%
25	870	00:19:58	99.23%	98.80%
30	1080	00:22:31	99.45%	99.20%

**FIGURE 20.**
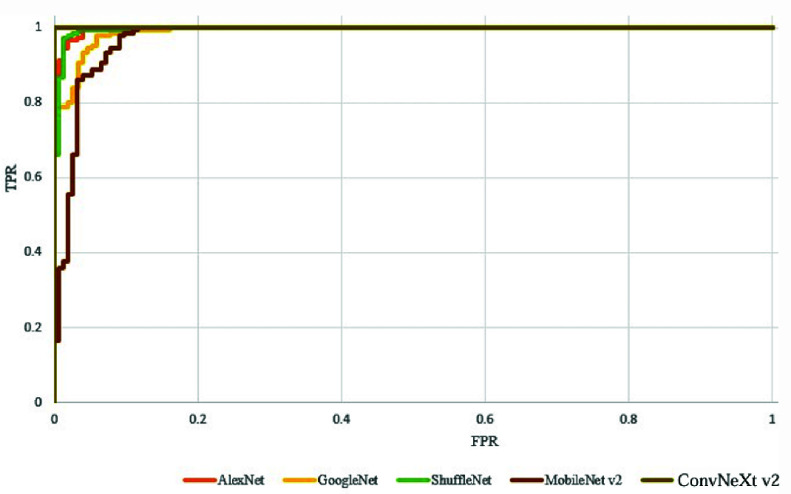
ROC curve used in this study.

The training results incorporating masking and image enhancement are presented in Table 6. AlexNet achieves the best training performance on both original and enhanced images, with accuracies of 93.36% and 95.59%, respectively. However, their differences are minor, and in some cases, only using image enhancement method decreases classification accuracy, particularly with ConvNeXt_v2. MobileNet v2 achieves the highest accuracy of 98.18% on masked images, while other CNN models also achieve training accuracies exceeding 95%. To further evaluate the model’s performance, we incorporate additional metrics beyond accuracy, including sensitivity (recall), specificity, and F1-score, as shown in Table 7. The results indicate that GoogleNet achieves the highest sensitivity (99.20%), while ConvNeXt_v2 demonstrates the highest specificity (98.48%) and F1-score (99.29%). Additionally, AlexNet, despite achieving the lowest accuracy (97.61%), maintains a competitive F1-score of 97.18%. A detailed comparison of the different methods reveals that masking and enhancement methods significantly improve classification performance. For instance, combining enhancement and masking with ConvNeXt_v2 achieves the highest accuracy (98.45%). It also results in the most extended runtime (19 min 12 sec) and higher memory consumption (75.48%), as seen in Table 7. However, despite its higher runtime and memory usage, ConvNeXt_v2 maintains the shortest inference time (0.271 sec), making it a highly efficient option for deployment in real-time applications. The training ROC curve is illustrated in Fig. 20. These results demonstrate that combining different methods effectively enhances the classification accuracy of classification models.

**TABLE 6:** Accuracy comparison between original and image enhancement

Model	AlexNet	Google Net	Shuffle Net	Mobile Net_v2	Conv NeXt_v2
Original	93.36%	92.55%	91.21%	91.43%	91.27%
Enhancement	95.59%	90.44%	92.66%	91.35%	88.70%
Masking	95.14%	95.76%	95.51%	98.18%	96.27%
Enhancement with masking	97.61%	98.41%	97.61%	97.86%	98.45%

**TABLE 7:** Enhancement combined with masking method training model accuracy and runtime

Model	Alex Net	Google Net	Shuffle Net	Mobile Net_v2	ConvNeXt_v2
Accuracy	97.61%	98.41%	97.61%	97.86%	98.45%
Sensitivity	96.80%	99.20%	98.40%	99.20%	99.90%
Specificity	97.58%	97.63%	97.61%	98.41%	98.48%
F1-Score	97.18%	98.41%	98.00%	98.80%	99.29%
Runtime (min: sec)	3: 22	3: 53	5: 33	7: 40	19: 12
Inference Time (sec)	0.345	0.382	0.273	0.412	0.271
Memory Usage	71.66%	65.00%	63.33%	70.83%	75.48%

This study integrates various image processing methods to improve robustness and enhance the AI model’s classification capability in evaluating third molar impaction on the IAN. Achieving high diagnostic accuracy in clinical applications ensures better healthcare quality while maintaining strong model performance on unseen data. This study has incorporated a Fuzzy voting mechanism to address potential overfitting and enhance model stability and generalizability [34]. This method aggregates the predictions of five models, reducing misclassification and ensuring that the final decision reflects the uncertainty levels across different models. This study assigns five levels of weights to the models, ranked from best to worst based on Accuracy. When the model’s predictions are closer to the others, they are considered more stable in their judgment and are assigned higher weights. We first compute the total absolute difference between each predicted value and all others to represent its dissimilarity; the reciprocal of this value serves as the initial similarity score and is defined in (8). These scores are then normalized so that their sum equals 1, which is defined in (9), and the resulting weights are used to compute the final aggregated accuracy. This approach emphasizes models being more consistent with the majority and suppresses those that deviate from the consensus.

\begin{align*} s_{i}& = \frac {1}{\sum \nolimits _{\begin{array}{l} j=1 \\ j\ne i \\ \end{array}}^{n} \left |{{ x_{i}-x_{j} }}\right |} \tag {8}\\ w_{i}& = \frac {s_{i}}{\sum \nolimits _{k=1}^{n} s_{k}} \tag {9}\end{align*}

**FIGURE 21.**
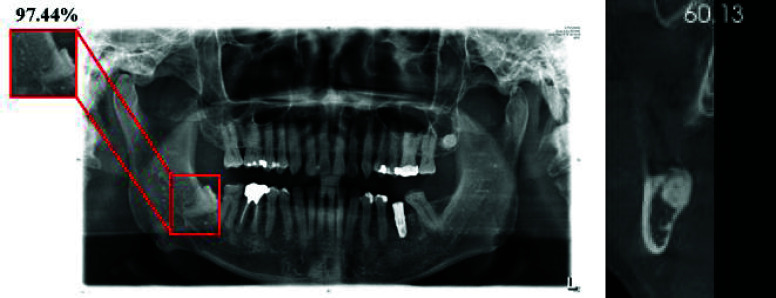
The validation results with DPR and the CT image for supplementary assessment.

As shown in Fig. 21, the validation images consist of DPR and CT scans, where CT imaging serves as a secondary diagnostic tool when DPR fails to provide a precise assessment. The CT scan in the figure reveals IAN compression by the third molar, confirming the clinical necessity for precise AI-assisted evaluation. Through the Fuzzy Voting mechanism, our model estimates a 97.44% probability of IAN compression in this case, demonstrating the effectiveness of our integrated approach in improving classification reliability. Furthermore, to verify the model’s stability and generalizability on external datasets, we randomly selected 100 DPR images from our dataset and the Tufts Dental Database [28] for accuracy evaluation. The validation results, presented in Table 8, demonstrate that the average accuracy of our dataset is approximately 97%. In comparison, the average accuracy on the Tufts Dental Database is around 94%, resulting in a 3% accuracy drop when tested on an external dataset. However, as the table shows, ConvNeXt_v2 consistently achieves the highest accuracy, reaching 97.71% on our dataset and 95.88% on the Tufts Dental Database.

**TABLE 8:** Validation comparison with Tufts dental database

Model	Alex Net	Google Net	Shuffle Net	Mobile Net_v2	Conv NeXt_v2
This study	96.88%	97.35%	96.74%	96.49%	97.71%
Tufts [28]	93.79%	93.53%	94.41%	94.72%	95.88%

## IV. Discussion

The third molar tooth positioning is a critical aspect of dental medicine and is frequently explored within artificial intelligence and dental science. This study employs two distinct methodologies to determine the position of the third molar: the application of advanced object detection models such as YOLO and a novel edge region segmentation algorithm explicitly developed for perioral tooth segmentation. The accuracy achieved by these methods were 95.86% and 97.92%, respectively. Furthermore, this study utilizes multiple analytical methods to assess these methods, with comparative results in Table 7. The findings indicate that the current research improves the accuracy of third molar positioning by at least 3% over previous research, highlighting the efficacy of integrating advanced computational strategies in dental diagnostics.

The third molar positioning system designed in this study incorporates enhanced methods for the IAN and symptom analysis using the ConvNext_v2 model, which is based on the Transformer architecture. This integrated approach offers an innovative technological pathway for improving the accuracy of third molar positioning and diagnosis. This study presents an advanced third molar localization system, integrating IAN enhancement methods with a Transformer-based ConvNeXt_v2 model for symptom analysis. This multi-technology fusion strategy offers an innovative approach to improving third molar localization and diagnosis accuracy. Table 10 provides a comparative analysis of existing studies on IAN compression detection, demonstrating the superior performance of our approach. For example, we compared with Chen et al. [17], which achieved the highest accuracy among prior studies. This study utilized the same number dataset size of 500 annotated DPR images and applied respective image enhancement methods for model training to ensure a fair evaluation. The results indicate that our Transformer-based ConvNeXt_v2 model outperforms AlexNet used in [17], yielding an accuracy improvement of approximately 4.5%, providing more precise and reliable auxiliary decision-making tools for clinical applications.

**TABLE 9:** Comparison in third molar tooth position detect accuracy

This study	YOLOv10 with image enhancement		95.86%
	Edge region segmentation algorithm		97.92%
Shin et al. [12]	Chen et al. [13]	Chen et al. [18]	Fukuda et al. [23]
94.3%	93.28%	94.18%	87.2%
Huang et al. [35]	Kim et al. [36]	Li et al. [37]	Zhou et al. [38]
92.78%	91.7%	91.13%	88.59%

**TABLE 10:** Comparison in third molar compress ian detect accuracy

Method	Model	Accuracy
This study	ConvNeXt_v2	98.45%
Chen et al [17]	AlexNet	93.90%
Fukuda et al. [23]	VGG-16	88.06%
Jeon et al [24]	EfficientDet	78.65%
Joo et al [39]	DenseNet	87.80%

However, this study still faces several challenges. The current DPR dataset does not fully generalize to all DPR imaging conditions and clinical scenarios. Although we incorporated the Tufts Dental Database [28] as an external validation dataset, it is still insufficient to represent the global diversity of oral characteristics across different populations. Future studies should expand the dataset to include a broader range of DPR images from diverse demographics, imaging equipment, and clinical conditions to improve model robustness. Additionally, while our results demonstrate that the model achieves an accuracy exceeding 95%, maintaining such high accuracy raises concerns about overfitting. Overfitting can arise from variations in image quality and dataset size differences, necessitating appropriate parameter adjustments to ensure model generalizability. Furthermore, as evidenced in the Results section, we observed that Transformer-based models require significantly more computational time than CNN-based models. This highlights a trade-off between accuracy and computational efficiency. Future research will explore model quantization and pruning strategies to optimize inference speed while maintaining diagnostic accuracy, enhancing the feasibility of Transformer-based approaches for real-time clinical applications.

## V. Conclusion

The primary objective of this study is to utilize DPR for image processing and classify whether the third molar compresses the IAN, aiming to reduce the demand for CT images in symptom diagnosis and mitigate the risks associated with high-dose radiation. A region segmentation method for DPR is proposed and achieves an accuracy of 97.92%. Subsequently, by introducing an edge masking technique for third molar images, the classification accuracy of whether the third molar compresses the IAN is enhanced, with a maximum accuracy of 98.45%. Future work will highlight features of the neural canal, which is anticipated to be a pivotal issue in related research fields. Additionally, adjustments and optimizations of the classification model are expected to enhance its efficiency. The findings of this study are poised to contribute to the field of third molar dentistry, thereby improving medical efficiency and enhancing doctor-patient relationships.
